# Accelerated Partial Breast Irradiation (APBI): A review of available techniques

**DOI:** 10.1186/1748-717X-5-90

**Published:** 2010-10-04

**Authors:** Christopher F Njeh, Mark W Saunders, Christian M Langton

**Affiliations:** 1 Radiation Oncology Department, Texas Oncology Tyler, 910 East Houston Street, Tyler, Texas, USA; 2 Physics, Faculty of Science and Technology, Queensland University of Technology, Brisbane, Australia

## Abstract

Breast conservation therapy (BCT) is the procedure of choice for the management of the early stage breast cancer. However, its utilization has not been maximized because of logistics issues associated with the protracted treatment involved with the radiation treatment. Accelerated Partial Breast Irradiation (APBI) is an approach that treats only the lumpectomy bed plus a 1-2 cm margin, rather than the whole breast. Hence because of the small volume of irradiation a higher dose can be delivered in a shorter period of time. There has been growing interest for APBI and various approaches have been developed under phase I-III clinical studies; these include multicatheter interstitial brachytherapy, balloon catheter brachytherapy, conformal external beam radiation therapy and intra-operative radiation therapy (IORT). Balloon-based brachytherapy approaches include Mammosite, Axxent electronic brachytherapy and Contura, Hybrid brachytherapy devices include SAVI and ClearPath. This paper reviews the different techniques, identifying the weaknesses and strength of each approach and proposes a direction for future research and development. It is evident that APBI will play a role in the management of a selected group of early breast cancer. However, the relative role of the different techniques is yet to be clearly identified.

## Introduction

Breast cancer is a worldwide problem, accounting for 10.4% of all cancer incidence among women, making it the second most common type of non-skin cancer (after lung cancer) and the fifth most common cause of cancer death. In the USA, breast cancer has the highest incidence among all cancer types in females with one in every eight to ten women being affected during her lifetime [1]; it is estimated that 192,370 women will be diagnosed with, and 40,170 women will die of, cancer of the breast in 2009 [2-4].

Breast cancer is the most common cancer in the UK among women although it is rare in men. In 2006 there were 45,822 new cases of breast cancer diagnosed in the UK: 45,508 (over 99%) in women and 314 (less than 1%) in men. Breast cancer is by far the commonest cancer in women in the UK accounting for 31% of all cases. The next most common cancer in women is lung cancer, with 16,647 cases (11% of total) in 2006. So nearly, a third of all new cancers in women are breast cancers. It has been estimated that the lifetime risk of developing breast cancer is 1 in 1,014 for men and 1 in 9 for women in the UK. These were calculated using incidence and mortality data for 2001-2005 [5].

Early stage breast cancer is defined as stage II or less; on the basis of the lack of lymph node, metastasis and clinical lesion size of 2 cm or less [6]. The 'surveillance, epidemiology and end results' (SEER) program reported that in 2006, 60% of diagnosed breast cancers are early stage [2,3]. Similarly in Japan, the fraction of early stage breast cancer was reported to be 40.6% in 1996 [6]. With the increasing use of breast cancer screening by mammography, more and more patients will have their breast cancer diagnosed at the early stage. Hence, there is a need for proper clinical management of early stage breast cancer is required. Most women who are newly diagnosed with early-stage breast cancer have a choice of: breast-conserving surgery (such as lumpectomy), a mastectomy (also called a modified radical mastectomy), radiation therapy and systemic treatments.

## Rationale for Breast Conservation

Breast conservation therapy (BCT) is the procedure of choice for the management of the early stage breast cancer. BCT consists of resection of the primary breast tumor (lumpectomy, segmental mastectomy or wide local excision) followed by whole breast irradiation (WBI). A total dose of 45-50 Gy is delivered to the entire breast over 5 to 6 weeks (1.8 to 2 Gy per fraction). In most patients, a boost dose of 10-16 Gy to the tumor bed is added. The establishment of BCT as the standard of care resulted from many years of prospective studies such as the National Surgical Adjuvant Breast and Bowel Project (NSABP) B-06 studies [7-9]. These studies found equivalent survival and local control rates among women treated with BCT compared to those treated with mastectomy.

The value of radiation therapy as a breast conservation component has been further validated by studies comparing lumpectomy alone to lumpectomy and radiation therapy. These studies demonstrate a threefold reduction in recurrence with the use of radiation therapy following breast conserving surgery [7,10-13]. For patients with ductal carcinoma in situ (DCIS), randomized studies comparing lumpectomy alone to lumpectomy plus radiation therapy, conducted by the NSABP and European organization for research and treatment of cancer (EORTC) found a 55% and 47% reduction in the ipsilateral breast cancer events respectively, with the addition of radiation therapy [13,14]. These and other studies have been recently pooled-analysed by Clarke et al. [11] and Vinh-Hung et al. [12]. Vinh-Hung's analysis found that the relative risk of ipsilateral breast tumor recurrence after breast-conserving surgery, comparing patients treated with or without radiation therapy, was 3.00 (95% confidence interval [CI] = 2.65 to 3.40). Further, the relative risk of mortality was 1.086 (95% CI = 1.003 to 1.175), corresponding to an estimated 8.6% (95% CI = 0.3% to 17.5%) relative excess mortality if radiation therapy was omitted. BCT is well tolerated with minimal long-term complications, favorable cosmetic outcome and reduced psychological trauma [7,9]. Radiation therapy therefore is an essential component of BCT. It not only decreases local recurrence but improves overall survival [11,12]. Because of these excellent results and the better cosmetic outcome, the United States National Institute of Health released a consensus statement, recommending breast conserving treatment as the preferable option for women with early-stage breast cancer [15].

## Rationale for Accelerated Partial Breast Irradiation (APBI)

Despite the advantages of BCT, its utilization remains a problem [16]. It has been reported that many women who are candidates for BCT do not receive it, only 10% to 80% of patients actually receive it [17-19]. In addition 15% to 30% of patients who undergo lumpectomy do not receive radiation therapy [20-22]. Similarly in Japan radiation therapy is performed in approximately 70% of patients following breast conservation surgery [23]. The under utilization of BCT has been associated with the fact that some women cannot, or will not, commit to the usual 6- 7 week course of adjunct conventional radiation therapy that is part of the BCT package [24]. It has been further hypothesized that convenience, access, cost and other logistical issues are major contributing factors. Other logistical issues include: distance from the radiation therapy facility, lack of transportation, lack of social support structure and poor ambulatory status of the patient [18,25,26]. Other reasons that may steer women away from BCT that have been identified include physician bias, patient age and fear of radiation treatments [22]. There has been a desire therefore to identify a subset of women who may not benefit from the addition of radiation therapy after lumpectomy for early stage breast cancer; however, no such subset of women has been identified [27].

Another criticism of BCT relates to consumption of resources; while radiation therapy facilities in the USA have largely kept up with demand for post-lumpectomy radiation therapy, breast irradiation may constitute 25%-30% of patient visits and can stress a health-care delivery system. However, not all countries have such adequate resources. For example Palacios Eito et al. [28] reported that the number of external irradiation units available in Spain in 2004 (177) was clearly lower than the number desirable (266-316). There is significant shortage of radiation therapy equipment in most of Asia and pacific regions [29], Latin America [30], Africa [31] and Eastern Europe [32]. In Africa, the actual supply of megavoltage radiation therapy machines (cobalt or linear accelerator) was only 155 in 2002, 18% of the estimated need [31]. In 12 Asia-Pacific countries with available data, 1147 megavoltage machines were available for an estimated demand of nearly 4000 megavoltage machines [32].

The question that arises therefore is 'can similar rates of local control be achieved with radiation therapy delivered only to the area at highest risk for recurrence?' If so, radiation could be delivered in a significantly shortened period, thereby potentially making the BCT option available and attractive to more women. This is the concept of accelerated partial breast irradiation (APBI) [26,33,34].

The stronger case for APBI has come from both retrospective and prospective studies; reporting that 44% to 86% of local recurrence occurs close to the tumor bed [10,35-37]. Ipsilateral breast recurrences in areas other than the tumor bed occurred rarely in 3% to 4% of the cases [34]. An update of the NSABP B-06 trial also confirmed this pattern of local recurrence, with 75% of recurrences at, or near, the lumpectomy site with other site ipsilateral breast recurrence rates similar to the recurrence of contra-lateral second primary breast cancer [38]. Based upon this evidence, BCT, with whole breast irradiation has been criticized as an over-treatment. Whole breast treatments incorporate the entire breast (including the surgical cavity), overlying skin, lower axilla and even small portions of the heart and lung in the treatment fields; this may introduce avoidable toxicity [39] whereas partial breast irradiation spares more normal tissue.

An additional theoretical advantage of APBI is a decreased dose to normal tissue. With a smaller target volume, it may be expected that adjacent organs such as the heart and lungs will receive less radiation. Radiation-induced lung injury after treatment for breast cancer, such as pneumonitis, lung fibrosis and pulmonary function test changes, are well documented in the literature [40,41]. An increase in lung cancer incidence and mortality after irradiation for breast cancer has also been reported in large studies [42-45]. It worth noting that the increase risk of long-term cardiac-related mortality after BCT may not be significant with modern breast radiation therapy.

A number of pathology studies have also researched local breast recurrence [46,47]. In the study by Holland et al., mastectomy specimens from more than 300 women diagnosed with invasive breast carcinoma, who fulfilled the criteria for breast conserving therapy, were systematically investigated [47]. They found that of the 282 invasive cancers, 105 (37%) showed no tumor foci in the mastectomy specimen around the reference mass. In 56 cases (20%) tumor foci were present within 2 cm, and in 121 cases (43%) the tumor was found more than 2 cm from the reference tumor [47]. This study justified the concept that whole-breast treatment either with surgery or radiation therapy is necessary to achieve local control. Supporters of APBI argue that this study was flawed in its patient selection and that the quality of mammography used at the time may have missed radiographic evidence of multicentric disease that would today be detected [48]. Contrary to Holland's data, recent studies from women considered appropriate for breast-conservation therapy reveal that the microscopic extension of malignant cells is unlikely to be beyond 1 cm [49-51].

## Accelerated Partial Breast Irradiation (APBI) Techniques

APBI is an approach that treats only the lumpectomy bed plus a 1-2 cm margin, rather than the whole breast. By increasing the radiation fraction size and decreasing the target volume, this technique allows the treatment to be accomplished in a shorter period. APBI is generally defined as radiation therapy that uses daily fraction doses greater than 2 Gy delivered in less than 5 weeks. There are a number of approaches now available for the implementation of APBI, these include: multi-catheter interstitial brachytherapy, balloon catheter brachytherapy, 3D-CRT (conformal radiation therapy) and intra-operative radiation therapy (IORT). Each of these techniques is vastly different from one another in terms of degree of invasiveness, radiation delivery, operator proficiency, acceptance between radiation oncologist and length of treatment. It is important to review the basic principles of radiobiology, as well as critical aspects of patient selection, surgical endpoints and radiotherapy treatment planning. This paper reviews the different techniques, identifying the weaknesses and strength of each approach and proposes a direction for future research and development.

### Multi-catheter Interstitial Brachytherapy (MIB) Treatment Technique

**Figure 1:**
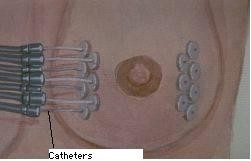
**Diagrammatic illustration of multi-catheter interstitial brachytherapy**.

Multi-catheter interstitial brachytherapy is the APBI technique that has been utilized the longest and has the most extensive follow-up [24,33,52]. This technique was initially developed to provide boost radiation after whole breast radiation therapy. Flexible after-loading catheters are placed through the breast tissues surrounding the lumpectomy. The catheters are inserted at 1 to 1.5 cm intervals in several planes; firstly to ensure adequate coverage of the lumpectomy cavity plus margins (Figure 1), and secondly, to avoid hot and cold spots. The procedure routinely requires between 14 to 20 catheters to assure proper dose coverage; the exact number being determined by the size and shape of the target, determined using established brachytherapy dosimetric guidelines [53,54].

Multiple catheters are placed in the breast using a free-hand or template-guided approach. The configuration of the catheters and their relation to the tumor target volume are crucial for effective treatment. Catheter insertion requires a high level of experience to produce an implant of excellent quality. The incorporation computed tomography (CT) based 3D planning and image-guidance has made a significant impact on the quality of the implants [55]. Determination of optimal catheter configuration prior to the procedure (virtual planning) would reduce the dependence of implant quality on the expertise of the physician [56].

In MIB either low dose rate (LDR) or high dose rate (HDR) brachytherapy may be used. With LDR, sources of Ir-192 sources are implanted for approximately 2 to 5 days while the patient is admitted as an inpatient. HDR however is an outpatient procedure, fractionated over the course of a week, with each treatment varying between seconds to minutes. The proposed dose of 34 Gy in 10 fractions BID (twice daily) for HDR was based on equivalence of the BED (biological effective dose) of this schema to 45 Gy in 4.5 days of the LDR regimen used in early APBI trials [57].

The majority of APBI patients treated with the longest follow-up have been treated with multi-catheter interstitial brachytherapy. A systematic review of these experiences have recently been presented by Offersen et al. [52]. Polgar et al. [58] and Antonucci et al. [59] have recently reported 12 year and 10 year follow-up respectively. In the study of Antonucci et al., eight ipsilateral breast tumor recurrences (IBTRs) were observed in patients treated with MIB resulting in a 10 years cumulative incidence of 5% (95% confidence interval [CI] 1.5-8.5%). The rate of incidence for WBI was 4% (95% CI: 1.3-6.7%), which not statistically significantly different from MIB treated patients. Table 1 presents some of the reported MIB studies with more than 5 years follow up.

**Table 1:** Results of recent clinical experience with Interstitial brachytherapy with more than 5 years follow up

Author	No of cases	Follow up interval (years)	Dose rate/pt no	Scheme	Total dose (Gy)	5-year LR (%)	Good/Excellent cosmesis
Strnad et al.[60]	274	5.25	PDR/HDR	PDR = 0.6 Gy/hr HDR = 4 Gy x8	PDR = 50 Gy HDR = 32 Gy	2.9%	90%

Antonucci et al. [59]	199	9.6	LDR/HDR	LDR 0.52 Gy/h × 96 hours HDR = 4 Gy x8 HDR = 3.4 Gyx10	LDR = 50 Gy HDR = 32 Gy HDR = 34 Gy	5%	99%

Johansson et al.[61]	50	7.2	PDR	50Gy/5	50	4%	56%

Arthur et al.[62]	99	7	LDR/HDR	LDR = 3.5 -5 days HDR = 3.4 × 10	45 Gy (LDR) 34 Gy (HDR	4%	n/a

Polgar et al.[63]	128	6.8	HDR	5.2 × 7	36.4 Gy	4.7%	77%

King et al [64]	51	6.25	LDR/HDR	LDR = 4 days 4 Gyx8	45 Gy (LDR) 32 Gy (HDR)	3.9%	75%

Otto et al. [65]	274	5.25	PDR/HDR	PDR 5 days, 0.6 Gy/hr HDR = 4 Gyx8	49.8 Gy (PDR) 32 Gy (HDR)	2.9%	92%

Polgar et al.[58]	45	11.1	HDR	4.33 × 7 5.2 × 7	30.3 Gy 36.4 Gy	4.4%	78%

LR = local recurrence, HDR = high dose rate, LDR = low dose rate, PDR = pulsed dose rate, n/a = data not available

### Balloon-Based Brachytherapy Devices

The balloon based brachytherapy include Mammosite, Axxent electronic brachytherapy, and Contura.

#### 1 MammoSite

**Figure 2:**
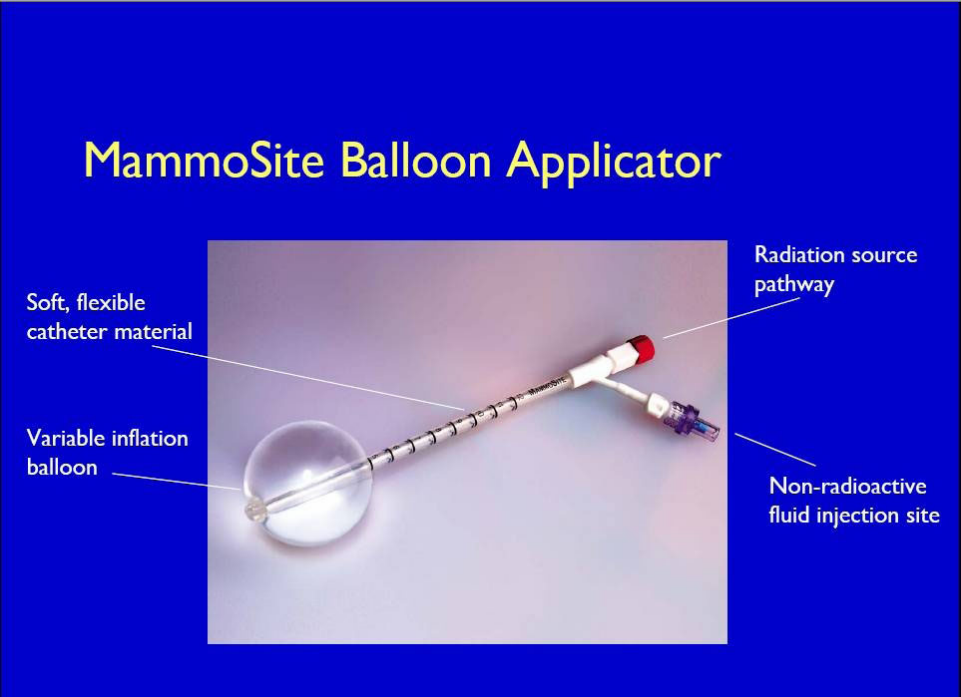
**The MammoSite Balloon applicator (courtesy of Hologic, Marlborough)**.

Although MIB has had very encouraging results, the technical challenges limit its widespread application. The MammoSite^® ^brachytherapy (MSB) system (Hologic, Marlborough, MA) applicator was developed to be more reproducible, easily applied and better tolerated. The mammosite catheter consists of a silicone balloon connected to a 15 cm double-lumen catheter (Figure 2) that is 6 mm in diameter. The catheter has both a small inflation channel and a channel for the passage of an Ir-192 high dose rate (HDR) brachytherapy source. The source channel runs centrally through the length of the balloon. The balloon is inflated with saline solution mixed with a small amount of contrast material to aid visualization. The balloon is inflated to a size that would completely fill the lumpectomy cavity and ensures conformance of the tissue to the balloon. An Ir-192 radioactive source, connected to a computer-controlled HDR remote after-loader, is inserted through the catheter into the balloon to deliver the prescription radiation dose [66,67].

**Figure 3:**
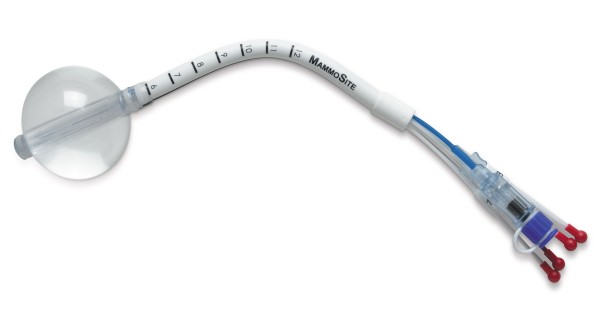
**The MammoSite Multilumen System (courtesy of Hologic, Marlborough)**.

The MammoSite applicator can be placed into the lumpectomy cavity at the time of surgery or in a separate procedure after surgery. In the latter case, the applicator can be inserted under ultrasound guidance either through the lumpectomy scar or via small separate incision. Following placement, a computed tomography (CT) scan is performed to assess the quality of the implant and for use in radiation planning. Implant quality is determined by examination of three parameters: balloon conformance to the lumpectomy cavity, distance from the surface of the balloon to the skin surface, and the symmetry of the balloon in relationship to the central catheter. Treatment planning parameters are: the diameter of the inflated balloon, the planning target volume, and the dose distribution [66-68]. While a minimum balloon-to-skin distance of 5 mm is required, a threshold of at least 7 mm is strongly recommended [69,70]. A longer skin distance is associated with greater improvement in cosmesis [71]. Conformance of the balloon to the lumpectomy cavity is assessed by quantifying the volume of the planning target volume (PTV) that is filled by air or seroma fluid. Adequate conformance is considered to have been achieved when less than 10% of the PTV is composed of fluid or air. A symmetric implant in relation to the source channel is also essential for adequate dosimetry. A non-symmetrical implant can result in dose inhomogeneity in the surrounding tissues since the MSB device contains a single, central source channel that does not allow for shaping of the radiation isodose curves in the direction perpendicular to the central channel [67]. The MSB may not be suitable in patients with small breast or for tumors located in the upper-inner quadrant because of the requirement for skin-to-cavity distances. Recently, Hologic has introduced a MammoSite Multi-lumen (4 lumen) device with the potential to eliminate some of the drawbacks of the single lumen device (see figure 3)

The MSB radiation therapy device generally delivers 34 Gy over 10 fractions (3.4 Gy per fraction, twice daily (BID)). The prescription point is 1 cm from the balloon surface with a minimum of 6 hours between fractions on the same day.

The MSB was approved by the USA food and drug administration (FDA) in May of 2002 and September 2009, the multi-lumen device was also approved. Bensaleh et al [68] and Shah and Wazer [72] have recently reviewed the MSB system. There are limited published data regarding the long-term tumor control and cosmesis associated with MSB. However, the results thus far are promising. Some of the studies with more than 12 months follow up are presented in Table 2, with the longest follow up published by Benitez et al. [73]. In this study, 43 patients were treated with MSB and had a median follow up of 65 months. So far, no loco-regional recurrences have been identified, with cosmetic outcomes of good to excellent achieved in 81.3% of the patients. Toxicities were significantly less frequent in patients with skin spacing of greater than 7 mm. The American Society of Breast Surgeon (ASBS) [71] registry trial recently reported 1440 patients treated, with a median follow up of 30.1 months. There have been 23 cases (1.6%) of ipsilateral breast tumor recurrence for a two-year actuarial rate of 1.04%. The cosmetic outcome of good to excellent was 95% at 12 months. For a subset of patients (n = 194) with DCIS in the ASBS registry, 6 patients (3.1%) had an ipsilateral breast recurrence, with 1 (0.5%) experiencing recurrence in the breast and axilla, for a 5-year actuarial local recurrence rate of 3.39% [74]. The acute and late-term toxicity profiles of MSB have been acceptable. Cosmetic outcome is improved by proper patient selection and infection prevention [70].

**Table 2:** Results of some of the recent clinical experience with Mammosite Brachytherapy System with more than a year follow up.

Author	No of cases	Median follow up interval(months)	IBF	Good/Excellent cosmesis
Benitez et al.[73]	43	65	0%	81.3%

Niehoff et al [69]	11	20	0%	n/a

Patel et al.[75]	26	48.5	0%	n/a

Vicini et al.[71]	1440	30	1.6%	95%

Chen et al.[76]	70	26.1	5.7%	n/a

Belkacemi et al. [77]	25	13	0%	84%

Voth et al.[78]	55	24	3.6%	n/a

Dragun et al. [70]	90	24	2.2%	90%

Vicini et al.[79]	1440	60	2.6%	90.6%

Jeruss et al. [74]	194^$^	54.4	3.1%	92%

n/a data not available, IBF = ipsilateral breast failure, $ these are ductal carcinoma in situ (DCIS) patients recruited in the American Society of Breast Surgeons APBI registry trial

#### 2. Axxent Electronic Brachytherapy

**Figure 4:**
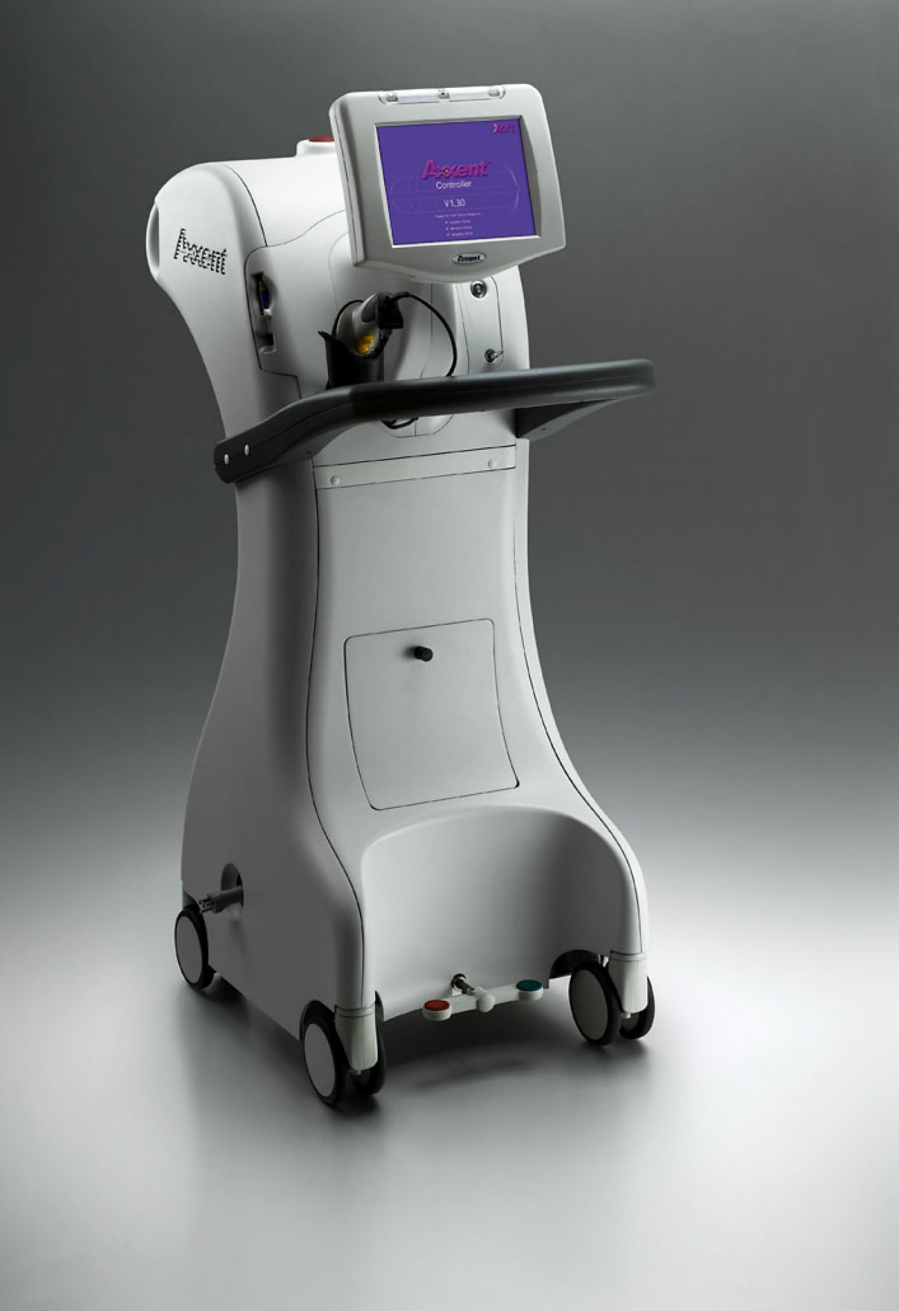
**Axxent electronic brachytherapy, controller front view (courtesy of Xoft)**.

**Figure 5:**
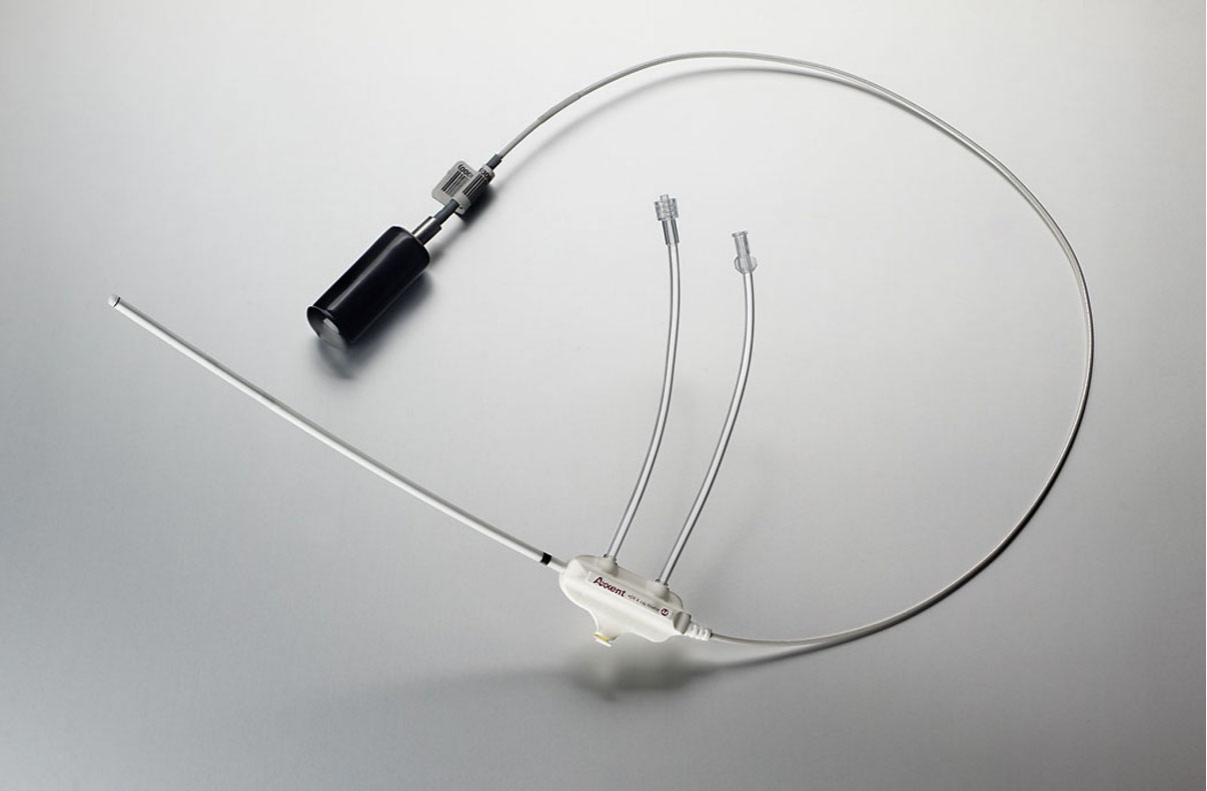
**Axxent electronic brachytherapy, HDR X-ray source (courtesy of Xoft)**.

**Figure 6:**
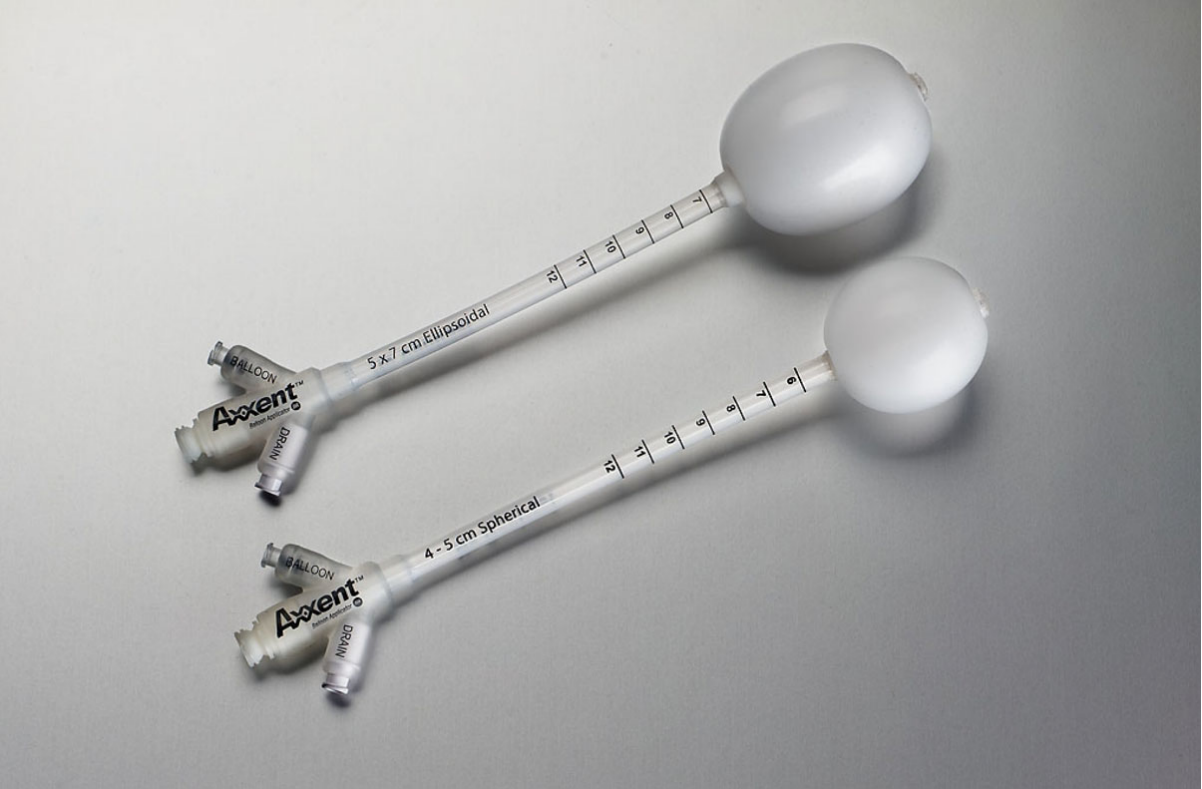
**Axxent electronic brachytherapy, balloon applicator (courtesy of Xoft)**.

Since the MSB has shown promising results, other forms of balloon-based brachytherapy have been developed. The novel Axxent electronic brachytherapy (eB) system (Xoft, Fremont, CA) is a modified form of balloon-based brachytherapy [67,80] (Figures 4, 5, 6). It is similar to the MammoSite system, consisting of a balloon catheter that is inserted into the lumpectomy cavity by means of a percutaneous approach. The catheter similarly has a central lumen through which the source is inserted. A second port enables inflation of the balloon with saline and a third port may be attached for drainage of seroma fluid or air surrounding the lumpectomy cavity. The wall of the balloon is covered in radiolucent material that is visible on a plain x-ray film or CT scan: addition of radiographic contrast is not therefore required. The Axxent electronic brachytherapy system is novel in that it uses an electronic 50 kilo-voltage x-ray source rather then an iridium-192 (^192^Ir) high-dose-rate (HDR) source. The X-ray source consists of a miniature x-ray tube that is inserted into the balloon catheter and delivers the radiation therapy to the patient. The eB controller is a portable unit, consisting of a digital touch-screen for the Physician and Physicist to input treatment data and monitor treatment progress [67].

This approach implies that a specifically shielded radiation room or an HDR afterloader unit are not required, both of which are needed for treatment with brachytherapy using Ir-192. The elimination of these requirements potentially open-up this APBI approach to a wider usage, particularly for patients who do not live in close proximity to a radiation center with a HDR after-loader unit. Since a shielded room is not required for treatment and the eB device is very portable, the number of setting in which the device can be used increases. It has also been suggested to use the device for intra-operative radiation therapy [81] and 11 patients have successfully had IORT using the eB device [82]. eB received FDA clearance for the treatment of breast cancer in January of 2006.

One inherent problem with MSB techniques is the high skin dose when the excision cavity is near the skin surface; that can result in late effect skin toxicity. The Axxent source model S7500 has pronounced anisotropy resulting in decreased dose at the proximal portion of the balloon [83]; this can be used as an advantage to optimize skin dose, particularly, if the cavity to skin distance is small. This anisotropy can also be accounted for by placing a dwell position outside the balloon surface along the proximal end of the catheter [67].

Being a relatively new device, there is a dearth in clinical experience and hence there are no clear recommendations on clinical use, for example, the surface-to-skin distance using electronic brachytherapy. Chen et al. recently reported a case report of radiation recall associated with the eB device and docetaxel administration [84]. They argued that the prescription of 34 cGy at 1 cm may result in a higher skin dose (when the skin to balloon distance is less than 1 cm) for eB because of the relatively higher fall off rate of the 50 KVp photon compared to Ir-192. The patient that they reported had a surface-to-skin distance of 7.5 mm, greater than the 7 mm MammoSite guideline. The calculated dose to the skin was approximately 537 cGy per fraction. If an ^192^Ir source had been used instead, the skin dose would have been approximately 470 cGy per fraction, corresponding to a relative dose increase for the electronic source of approximately 14%.

Another potential contributing factor is the increase in relative biologic effectiveness (RBE, the ratio of doses for photons of differing energies required to produce the same biologic effect) related to the lower energy of the photons emitted by the electronic brachytherapy source. It is well established that the biological effectiveness of low-energy photons is large compared with higher-energy gamma rays, because of the dominance of photoelectric absorption at low energies [85]. The RBE for a 40 kVp source (very similar to the Axxent photon spectrum) has been calculated to be 1.28 greater than. an ^192^Ir source [85]; hence, the dose from the 192-Ir source must therefore be 1.28 times greater than that of the low energy photon source to produce the same effect (e.g., skin ulceration).

#### 3. Contura

**Figure 7:**
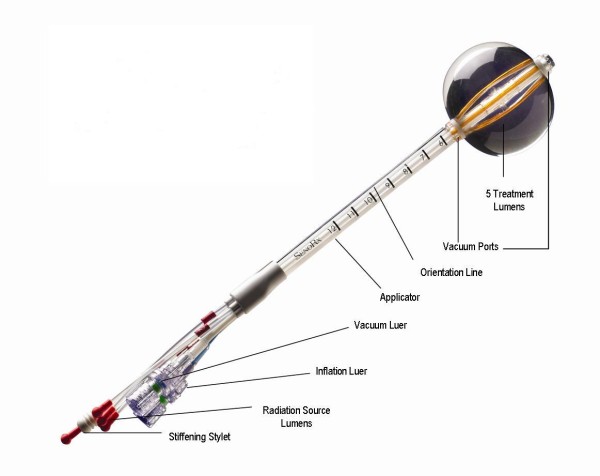
**The Contura balloon applicator (courtesy of SenoRx)**.

The balloon catheter of the Contura device (SenoRx, Inc, Aliso Viejo, Ca) differs from the MSB and eB catheters in that it has multiple lumens for passage of an Ir-192 HDR source (figure 7). In addition to a central lumen, the Contura balloon has four surrounding channels to accommodate the HDR source. The positions of the surrounding channels have a fixed 5-mm offset around the central channel [67]. These channels provide additional source positions and thus allow increased dose flexibility compared with a single-catheter approach. This approach has the potential to reduce the dose to normal tissues (chest wall and skin) and organs at risk such as the heart and lungs. In addition, multiple catheters make it possible to account for asymmetric balloon implant with respect to the central channel. Like the eB catheter, Contura has a port for a vacuum to remove fluid or air around the lumpectomy cavity; the use of this vacuum port can improve tissue-balloon conformance. The Contura device received FDA clearance in May 2007.

MSB has the longest duration in follow up and new APBI devices compare its clinical efficacy to that of MammoSite. A recent study by Wilder et al. [86] evaluated one hundred and eighty-two women with early breast carcinoma treated with post lumpectomy brachytherapy using Contura (n = 45) and MammoSite (n = 137) devices with a median follow-up of 16 months. A Contura catheter did not require explantation in 16% (7 of 45) of patients where balloon-to-skin spacing was only 3-6 mm and 11% (5 of 45) of patients where there was an air/fluid pocket greater than 10% of the planning target volume for plan evaluation. A MammoSite catheter was explanted in 10% of cases where the minimum balloon-to-skin distance was <7 mm and in 13% of cases where there was a large air/fluid pocket next to the balloon. They observed incidence rates of acute toxicity with a Contura device similar to those with a MammoSite device [86]. Brown et al. [87] have also reported similar improvements in dosimetric capabilities (i.e., reduced skin and rib doses and improved PTV_EVAL coverage) with the Contura device.

### Hybrid Brachytherapy Devices

Hybrid devices were developed to take advantages of the versatility and dosimetric conformity of multicatheter interstitial brachytherapy with the convenience and aesthetics of a single entry device. There are currently two devices in this category namely the Struts Adjusted Volume Implant (SAVI) and the ClearPath.

#### 1. Strut Adjusted Volume Implant (SAVI)

**Figure 8:**
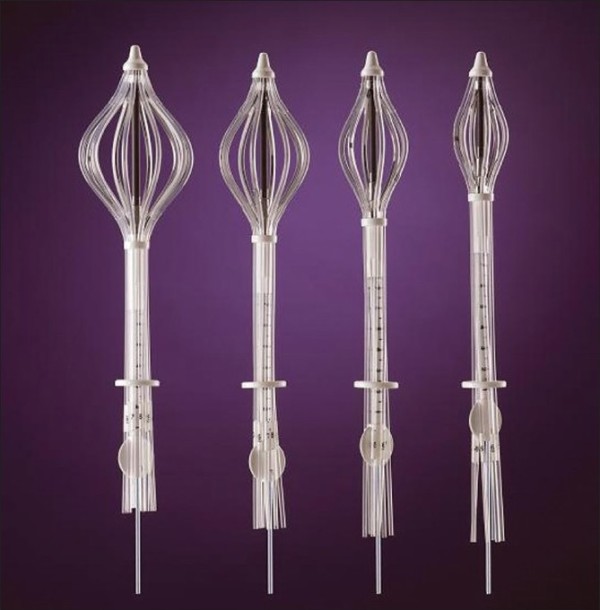
**Different sizes of SAVI with peripheral struts expanded (courtesy of Cianna Medical)**.

The SAVI device (Cianna Medical, Aliso, Viejo, Ca) (Figure 8) consists of a central strut surrounded by 6, 8 or 10 peripheral struts, depending on the size of the device [67,88]. The peripheral struts can be differentially loaded with a HDR source. The device is inserted in collapsed form through a small incision; once placed, it is then expanded to fit the lumpectomy cavity by clockwise rotation of a knurled knob at the proximal end of the expansion device, expanding the peripheral struts and providing a pressure fit [89]. The outward pressure exerted by the expanded struts pushes against the cavity walls securing the struts in place. Some tissue invagination between the struts has been observed during the course of the treatment. Radio-opaque markers are present on three of the peripheral struts (number 2, 4 and 6) for identification during the reconstruction process in treatment planning.

The SAVI device is surgically implanted on an outpatient basis by the treatment radiation oncologist using ultrasound guidance with the patient under local anesthesia. A CT scan is acquired immediately following the implant surgery, both for the verification of the proper deployment of the device, and for treatment planning. It was recommended by Scanderbeg et al [89], that although the device does not move independently to the body, one should always try to attain a position as close to the planned patient position due to breast deformation. They found a breast board to be best for patient setup because of its ease of setup and reproducibility.

#### 2. ClearPath (CP)

**Figure 9:**
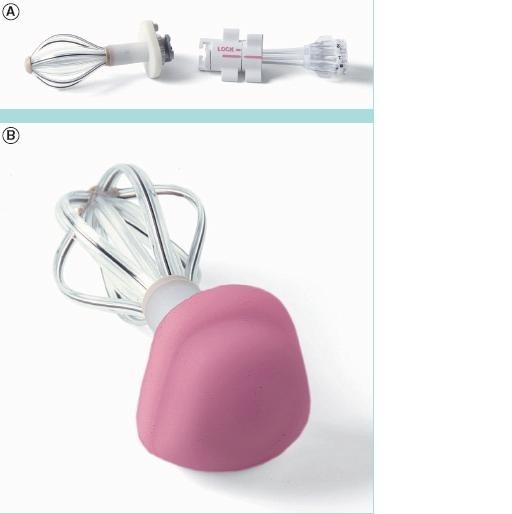
**ClearPath device (a) the base detached (b) a cap placed over the HDR channels (courtesy of North America Scientific)**.

Another hybrid device similar to the SAVI has also been developed called ClearPath (CP; North American Scientific (Chatsworth, CA)). CP was developed to combine the advantage of balloon brachytherapy and multicatheter brachytherapy. The CP consists of both inner and outer catheters that expand by rotating a knob on the base of the device (Figure 9) [67,90]. The CP device contains six outer expandable plastic tubes to displace the tissue. The radii of expansion of these tubes are adjusted at the base of the device and can be expanded to conform to a similar shape and size as a balloon device. In the center of the expandable tubes is a central catheter surrounded by six additional catheters that allow the passage of an HDR Iridium-192 source. In contrast to the SAVI device, the radiation source is not in direct contact with the breast tissue. In addition, after the device is placed in the patient, the rubber sleeve is sutured to the patient, and the base of the device is cut off. This leaves only the catheters exposed and visible external to the patient's skin [91]. Normally a cap is placed over the HDR channels. This could potentially lead to increased patient comfort by eliminating the dangling external catheters.

CP is a relatively new device and hence no clinical outcome data have been reported. However, retrospective dosimetric analysis has been reported [90,91]. Dickler et al. [91] found that MSB and CP offered comparable target volume coverage, but CP allowed significantly more normal-tissue sparing. Similarly, Beriwal et al. simulated a phantom study and the parameters of the CP catheter were superimposed on the MSB planning CT scans. The authors found that the median maximum skin dose was 161% for MSB and 113% for CP of the prescription dose [90].

### External Beam Radiation Therapy (EBRT)

**Figure 10:**
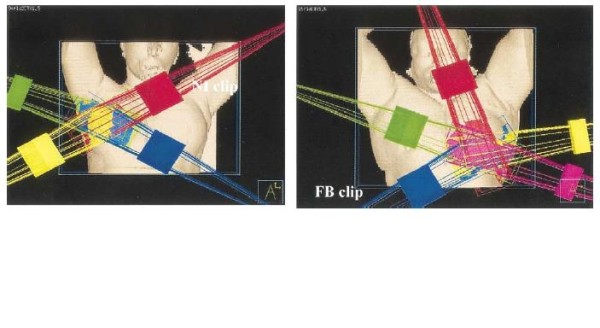
**3D-CRT typical 4-field arrangement for right sided lesions and 5 field arrangement for left sided lesions (reprinted with permission from Baglan et al**.[93].

Several techniques may be classified as 'external beam radiation therapy' including 3D-conformal radiation therapy (3D-CRT) with multiple static photons, and/or electrons fields, intensity modulated radiation therapy (IMRT) and proton beams [92]. The most widely used 3D-CRT approach was initially described by Baglan et al [93]. This technique was adopted for use as one of the allowed treatment modalities for patients randomized to APBI in the National Surgical Adjuvant Breast and Bowel Project B- 39/Radiation Therapy Oncology group (NSABP/RTOG) 0413 phase III trail [94]. The technique uses four to five tangentially positioned non-coplanar beams (Figure 10). The tumor bed is defined by the computed tomography visualized seroma cavity, postoperative changes, and surgical clips, when available. The clinical target volume (CTV) is defined as the tumor bed with a 1.5 cm margin limited by 0.5 cm from the skin and chest wall. The planning tumor volume (PTV) is defined as the CTV with a 1.0 cm margin. The prescription dose used for NSABP/RTOG protocol is 3.85 Gy twice daily (separated by at least 6 hours) to a total dose of 38.5 Gy delivered within 1 week [94].

EBRT has many potential advantages, over the other techniques [95].

1. The technique is non-invasive and the patient is not subjected to a second invasive surgical procedure or anesthesia, thereby reducing the potential risk of complications. The treatment can wait until completion of pathological analysis about the original tumor and the status of the resection margins are available.

2. The technique has potential for widespread availability since most radiation therapy centers already perform 3D-CRT for other cancers.

3. It is likely that an external beam approach will be easier for radiation oncologists to adopt than brachytherapy techniques because the technical demands and quality assurance issues are much simpler.

4. Treatment results with external beam may be more uniform between radiation oncologists because the outcome depends less on the experience and operative skills of the person performing the procedure than for brachytherapy (especially using interstitial implantation).

5. It seems less likely that technical issues arising during external beam radiation therapy will require the procedure to be aborted as is not infrequently the case when brachytherapy techniques are used.

6. External beam is intrinsically likely to generate better dose homogeneity and thus may results in a better cosmetic outcome when compared with bracytherapy techniques.

Despite the above appeal of EBRT APBI, many issues and unanswered question remain. These include breathing motion, treatment setups variation, and the fractionation scheme adopted. The target may move during breathing and the patient may be positioned differently for different fractions. To avoid missing the planned target, a large treatment volume is used. A prone patient position has been suggested by Formenti et al.[96] to minimize target tissue movement during breathing. The prone position also provides exceptional sparing of the heart and lung tissues. Unfortunately, the prone position is not widely used because it requires a special immobilization device and is uncomfortable for some patients.

The use of multiple treatment fields in 3D-CRT/IMRT can increase the volume of normal tissue irradiated to low or moderate doses (i.e increase in integral dose). Also, 3D-CRT delivers higher doses to normal breast tissue since the PTV around the lumpectomy cavity is increased to account to breathing and setup errors [97].

The identification and contouring of the lumpectomy cavity (LC) is another issue with 3D-CRT APBI. LC determination is critical because treatment delivery is delayed after breast surgery. Furthermore, the GTV and CTV are generally defined as the contouring of a seroma within the lumpectomy cavity, expanded by some margin, usually 1 cm [93]. However, the delineation of the seroma could vary among different observers and even among experienced ones [98]. It has been suggested by Dzhugasvili et al. [99] that the use of surgical clips as fudicial markers may reduce such observer variability.

There is still the question of the appropriate dose and fractional scheme for 3D-CRT APBI. As evident in Table 3 different doses and fractionation schemes have been reported in the literature. Rosenstein et al. [100] assessed the biologically equivalent doses (BEDs) of several APBI schedules using a linear quadratic model. Using an α/β ratio of 10, they found the Vicini fractionation scheme provided a BED of 53 Gy, the Formenti fractionation scheme gave 48 Gy and the 32-Gy dose used by Taghian et al. [101] gave a BED of 45. Livi et al.[102] in randomized Phase III trial have used a dose of 30 Gy in five fractions (6 Gy/fraction) and argued that it was equivalent to 54 Gy in a standard fractionation of 2 Gy fractionation. However, Cuttino et al. [103] utilizing a wide range of established radiobiological parameters, determined that the maximum fraction size needed to deliver a biologically equivalent dose using 3D-CRT is 3.82 Gy, supporting the continued use of 3.85Gy BID in the current national cooperative trial.

**Table 3:** Accelerated partial breast irradiation clinical studies using external beam radiation

Author	No of cases	Follow up (months)	Fractionation scheme	IBF	Good/Excellent cosmesis
Vicini et al[104]	52	54	3.85 Gy × 10 (bid)	6%	n/a

Vicini et al.[105]	91	24	3.85 Gy × 10 (bid)	0%	90%

Chen et al. [106]	94	51	3.85 Gy × 10 (bid)	1.1%	89%

Taghian et al.[107]	99	36	3.2 Gy × 4 (bid)^$^	2%	97%

Formenti et al.[108]	10	36 (minimum)	5.0, 5.5, 6.0 Gy × 5 (10 days)	0%	100%

Formenti et al.[96]	47	18	6.0 Gy x5 (10 days)	0%	n/a

Magee et al.[109]	353	96 (mean)	5.0 - 5.31 Gy × 8 (10 days)^&^	25%	n/a

Leonard et al. [110]	55	34 median	3.85 cGy x10 (bid)	0%	n/a

Hepel et al.[94]	60	15	3.85 Gy × 10 (bid)	n/a	81.7%

Jagsi et al.[111]	34	> 24	3.85 Gy × 10	n/a	79.5%

^$^Technique used were: mixed photons and electrons (63 patients), photons alone (16 patients), and protons (20), ^&^Technique was electron field with a beam energy of 8-14 MeV, the majority being treated with 10 MeV, IBF = ipsilateral breast failure, n/a = data not available.

### Intra-Operative Radiation Therapy Techniques

Intra-operative radiation therapy (IORT) refers to the delivery of a single fractional dose of irradiation directly to the tumor bed during surgery. These techniques have been reviewed by Reitsamer et al. [112], Vaidya et al. [113,114] and Orecchia and Veronesi [115]. Older intra-operative radiation therapy devices were technically cumbersome, commonly relying on the transportation of the patient from the operating theatre to the radiation therapy unit during surgery, or require custom-built intra-operative radiation therapy theatres [113]. These technical and financial limitations to delivery of intra-operative radiation therapy have prevented widespread use of the approach. Advances in miniaturization technology have enabled the development of mobile intra-operative radiation therapy devices. Intra-operative radiation therapy was first used in 1998 with a device called the Intrabeam, since then, two other mobile linear accelerators have become available (the Mobetron and Novac-7 systems). These systems either generate megavoltage electrons (Mobetron and Novac-7) or kilovoltage photons (intrabeam).

The potential advantages of IORT include delivering of the radiation before tumor cells have a chance to proliferate. Furthermore, tissues under surgical intervention have a rich vascularization, with aerobic metabolism, which makes them more sensitive to the action of the radiation (oxygen effect). Also, the radiation is delivered under direct visualization at the time of surgery. IORT could minimize some potential side effects since skin and the subcutaneous tissue can be displaced during the IORT to decrease dose to these structures, and the spread of irradiation to lung and heart is reduced significantly [116]. IORT eliminates the risk of patients not completing the prescribed course of breast radiotherapy (a well-recognized risk of conventional breast radiotherapy) and allows radiotherapy to be given without delaying administration of chemotherapy or hormonal therapy [117]. IORT has the potential for accurate dose delivery: by permitting delivery of the radiation dose directly to the surgical margins, IORT eliminates the risk of geographical miss in which the prescribed radiation dose is inaccurately and incompletely delivered to the tumor bed. Geographical miss may result from patient movement, inconsistent patient setup, and difficulty identifying the tumor site weeks or months postoperatively and is estimated to occur in up to 70% of patients receiving conventional breast boost radiotherapy [118]. There is potential for decreasing healthcare cost because it is one fraction as opposed to 25 fractions.

With IORT the final pathology reports arrives days post-festum. This has been one of the major criticisms of the technique. So recently a novel handheld probe (Dune Medical Devices, Caesarea, Israel) has been developed for intra-operative detection of positive margins [119] Such a device can help reduce re-excision rate and improve acceptance of IORT technique.

#### 1. INTRABEAM (X-rays)

**Figure 11:**
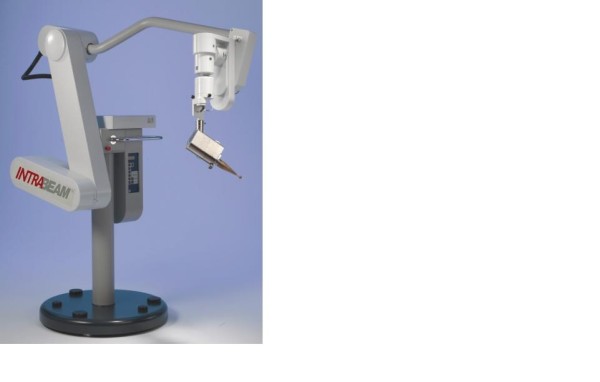
**The mobile X-ray intraoperative radiation therapy device: The Intrabeam device intraoperative photon device**.

**Figure 12:**
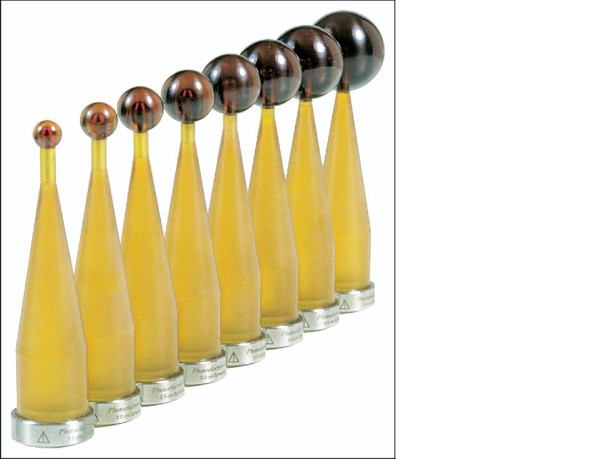
**Various spherical applicators with diameters ranging from 1.5 to 5 cm used in the intrabeam device (reprinted with permission Holmes et al: **[117].

The mobile X-ray system Intrabeam™ is manufactured by Carl Zeiss (Oberkochen, Germany) [120]. The system is composed of a miniature, light-weight (1.6 kg) X-ray source (PRS- 400), combined with a balanced floor stand with six degrees of freedom to gain access to target sites throughout the body (Figure 11). The miniature X-ray source has a probe of 10 cm length and 3.2 mm diameter. Within this device, electrons are accelerated to the desired energy level and focused down the probe to strike a gold target. Various spherical applicators with a diameter ranging from 1.5 to 5 cm are available to match the size of the surgical cavity (Figure 12). They are fixed to the end of the source and placed in the excision cavity to obtain a homogeneous dose distribution on the surface of the applicator and consequently on the surface of the tumor cavity. When mounted onto the Intrabeam unit, each spherical applicator conforms the breast tissue around the radiation source to permit delivery of a uniform field of radiation to a prescribed tissue depth. Accurate and uniform dose delivery is further achieved by placement of "pursestring" sutures within the breast to hold the pliable breast tissue against the applicator surface [117].

The X-ray system produces low-energy photons (30- 50 KVp) with a steep dose fall-off in soft-tissue; no special shielding is therefore required in the room [120]. Dosimetry varies by applicator tip size with the commonly used 3.5 cm applicator sphere delivering 20 Gy at a radius of 1 mm from the surface, 5 Gy at 10 mm and 1 Gy at 27 mm in about 20 minutes [113]. Treatment time lasts for approximately 20 to 45 minutes, depending on the size of the lumpectomy cavity, the size of the selected applicator, and the prescribed dose.

Treatment can be carried out in unmodified operating rooms with minimal exposure to the staff and patient; rapid dose fall-off in the tissue around the applicator guarantees minimal exposure of the surrounding tissue such as the lung and cardiac tissue in the patient.

The physics, radiobiology, dosimetry, and early clinical applications of this low energy x-ray device have been fully evaluated, and the device has received Federal Drug Administration approval for use in any part of the body since 1999 [121]. The RBE for this low-energy x-rays have been estimated to be 1.5 [85]. It has been suggested that the biologically weighted dose (physical dose × RBE) decreases with depth less quickly than physical dose [85] Therefore despite the steep gradient in physical dose, an effective uniform biological dose is distributed inside a rim of about 15 mm around the most often used intrabeam applicator [122]. Another potential advantage of Intrabeam is that, because normal tissues can repair their damaged DNA within a few minutes but cancer cells with poor DNA- repair machinery may be unable to repair quickly. So treatment given over a long time (intrabeam is between 25-35 minutes) may have a higher therapeutic index than giving similar doses over 2 to 3 minutes [114].

Encouraged by initial pilot studies (see Table 4), a phase III, prospective randomized non-inferiority trial called TARGIT (targeted intraoperative radiation therapy) began in March 2000. This trial compares single dose intraoperative radiation therapy targeted to the tumor bed to conventional whole breast external beam radiation therapy in early breast cancer.[114,117]. Patients were enrolled from 28 centers in nine countries including UK, Germany, Italy, USA and Australia. Data accrual was closed in May 2010 and the results of this trial have recently been published by Vaidya et al. [123]. In this trial 1113 patients were randomly assigned to the targeted intraoperative radiotherapy group and 1119 allocated to the whole breast external beam radiation therapy group. From this, 854 patients received targeted intraoperative radiotherapy, only 142 received targeted intraoperative radiotherapy with external beam radiotherapy and 1025 patients in the external beam radiotherapy group receiving the allocated treatment. They observed at 4 years follow up, there were six local recurrences in the intraoperative radiotherapy group and five in the external beam radiotherapy group. The Kaplan-Meier estimate of local recurrence in the conserved breast at 4 years was 1.20% (95% CI 0.53-2.71) in the targeted intraoperative radiotherapy and 0.95% (0.39-2.31) in the external beam radiotherapy group. The rate of recurrence between the two groups was not statistically significant. Similarly the total rate of major toxicities was similar in the two groups[123]. This study presents the first level 1 evidence of the equivalence of APBI using IORT to WBI and confirms that targeted IORT allows the entire dose of radiation therapy to be administered in a single fraction at the time of breast-conserving surgery, thus avoiding the need for repeated radiation therapy treatments or placement of in dwelling radiation therapy devices.

**Table 4:** Some clinical studies using Intra-operative radiation therapy (IORT).

Author	No of cases	Median follow up interval(months)	Technique	IBF	Good/Excellent cosmesis
Lemanski et al. [131]	42	30	Electrons	4.8%	100%

Veronesi et al.[130]	590	20	Electrons	0.5%	n/a

Mussari et al.[132]	47	48	Electrons	0%	92%

Vaidya et al.[121]	25	24	Photons	0%	n/a

Vaidya et al. [123]	854	48	photons	1.2% (95%CI = 0.53-2.71) ^$^	n/a

^$^TARGIT phase III trial, at 4 years there 6 local recurrences in the target treated group and 5 in the whole breast treated group, giving the Kaplan-Meier estimates (not crude estimates), n/a not available, IBF = ipsilateral breast failure.

#### 2. MOBETRON (electrons)

**Figure 13:**
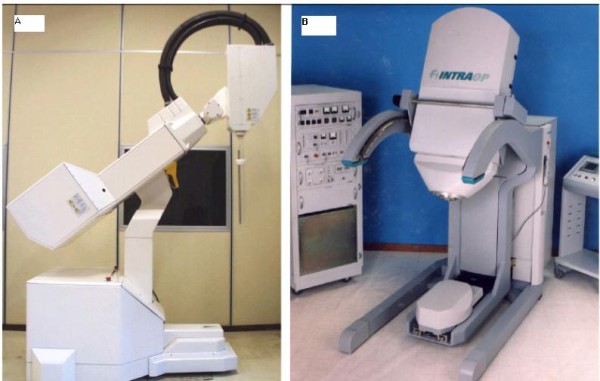
**The mobile electron intraoperative radiation therapy devices: (a) Novac7 (b) Mobetron intra-operative electron device (reprinted with permission Beddar el al**. [124]).

The Mobetron (IntraOP Medical Inc, Santa Clara, CA), is a mobile electron beam intraoperative treatment system. The Mobetron system (Figure 13a) is composed of three separate units: the control console, the modulator and the therapy module [124]. The control console which operates the accelerator during radiation treatment delivery is placed outside the OR so that the radiation treatment delivery is controlled remotely. The modulator houses the electronic systems of the accelerator and energizes the accelerator to produce the electron. The therapy module houses the accelerator guide and control systems that generate and deliver radiation [125]. The Mobetron uses two X-band (3 cm wavelength, 10 GHz frequency) collinear accelerators. This design eliminates the need for a bending magnet thus affecting a reduction in photon leakage [126]. The Mobetron system produces electrons of nominal energies of 4 MeV, 6 MeV, 9 MeV and 12 MeV with therapeutic ranges up to 4 cm. The system is designed to deliver a very large uniform dose of 10 to 25 Gy in a single fraction at a dose rate of 10 Gy/min [124].

#### 3. NOVAC-7 (electrons)

The NOVAC-7 system (Figure 13b) (Hitesys, Latina, Italy) delivers electrons with the use of a mobile dedicated linear accelerator; its radiating head can be moved by an articulated arm that can work in an existing operating room. It is based on a compact S-band standing wave electron beam linear accelerator utilizing a patented auto-focusing structure which eliminates the need of focusing solenoids. The accelerator is moved by six axis robotic arm. It delivers electron beams at four different nominal energies (3, 5, 7 and 9 Mev) [113]. Beam are collimated by means of a hard docking system, consisting of cylindrical perspex applicators available in different diameters (4 to 10 cm) and angles of the head (perpendicular or oblique 15° to 45° with respect to their axis).

A phase III, prospective randomized trial called ELIOT (electron intraoperative therapy) began in 2000 in Italy. A single dose of 21 Gy with energies up to 9 MeV, biologically equivalent to 58-60 Gy in standard fractionation is applied to the tumor bed. The dose of 21 Gy was established from a dose-escalating phaseI/II study [127]. The electron energy used is determined from the depth of the tissue to be irradiated. The accelerators used have been designed to have a dose rate (15-20 Gy per minute) higher than conventional and can deliver 21 Gy in less than 2 min [115]. The entire procedure last for about 15 to 20 minutes. Unnecessary radiation to the underlying normal tissue can be avoided by mobilizing the mammary gland during surgery and placing a lead plate for shielding on its dorsal surface.

The costs of the mobile linear accelerator with a robotic arm, used in intra-operative radiation therapy, are prohibitive for poor countries. Frasson et al. [128] evaluated the feasibility of ELIOT in the accelerator room of the radiation therapy service for early breast cancer treatment; demonstrating that intra-operative radiation therapy with electrons can be safely performed in an accelerator room with a conventional machine.

A systematic review by Cuncins-Hearn et al. [129] concluded that the short-term results were similar for both BCT and IORT in terms of local recurrence, disease-free and overall survival. The current evidence base is however poor, making definitive assessment on IORT very difficult. They suggested that further research is required to clarify several issues such as identification of the most appropriate subgroups of patients for IORT, a comparison of the currently available mobile IORT technologies, establishing whether IORT is most appropriate as a boost replacement dose or replacement for all postoperative radiation therapy, the examination of how biological repair processes may differ between the two treatment modalities and determining precisely where local recurrences originate with respect to the original tumor site.

The IORT approach has the advantage of shortening the treatment course further, conveniently delivering the entire course of local therapy at the time of initial excision. However, IORT also presents significant technical challenges, not the least due to the need for accuracy of target definition and treatment delivery inherent in a single dose radiotherapy delivery. One issue is the accuracy of tumor bed definition when tissues are re-approximated following excision. Another is the variable margin of normal tissue irradiated in the re-opposed tissues. IORT may be complicated in patients who are determined to have positive surgical margins and need re-excision. This may not have been a significant issue in the Versonesi studies [130], as all patients received generous resections with quadrantectomies.

## Discussion

The issue of the need and utility of APBI is highly debated within the medical community. There are those of the school of thought that the current standard of care for early breast cancer works well. So, the frame of mind is "if it is not broken why fix it?", evidenced by the commentary in medical journals such as "Is APBI a step backward?" [133]. On the other hand, there are those who belief that APBI has a role to play in the clinical management of early stage breast cancer [134]. If the proliferation of APBI techniques is anything to go by, there is indeed a high level of interest. As reviewed in this paper, there are several different approaches to APBI, each with their merits and limitations (Table 5, [135]). For those who believe in the role of APBI, there is a general consensus that a few questions remain to be satisfactorily addressed including the appropriate fractionation scheme, the appropriate patient selection criteria, the need for phase II/III clinical trials establishing equivalence or improvement to WBI and the appropriate technique.

**Table 5:** Comparison of the current available APBI techniques (adapted from Sarin [135]), MIB = multicatheter Interstitial brachytherapy, IORT = intraoperative radiation therapy, RCT = randomized Clinical trials, OAR organ at risk

	MIB	Balloon based brachytherapy			Hybrid based brachytherapy		External beam			IORT	
		**Mammosite**	**Axxent Electronic**	**Contura**	**SAVI**	**ClearPath**	**Photons**	**Electrons**	**Protons**	**electrons**	**Photons**

Prescription point	1.5 - 2 cm	1 cm	1 cm	1 cm	1 cm	1 cm	1.5 - 2 cm	1.5 - 2 cm	1.5-2 cm	10- 30 mm	2 mm

Coverage of target volume	Variable	Good	Good	Good	Good	Good	Best	Good	Best	Good	Good

Dose Homogeneity	Fair	Fair	Fair	Fair	Fair	Fair	Best	Fair	Best	Fair	Fair

Sparing of OAR	Good	Good	Better	Better	Better	Better	Least	Varies	Good	Good	Best

Skin Dose	Least	Variable	variable	variable	variable	variable	Least	maximum	Least	Least	Least

Expertise required	High	Average	Average	Average	Average	Average	Average	Least	High	Very High	High

Suitability for various tumor size, location and shape	Not suitable if inadequate tissue or near axilla	Not suitable for large/irregular cavities or at the periphery	Not suitable Large cavities	Not suitable Large cavities	Not suitable Large cavities	Not suitable Large cavities	May not be suitable for small breast	Not suited for deep seated cavities in large breast	Superficial tumor	Not suitable for tumors near brachial plexus/axilla or skin	Not suitable for large irregular cavities or at the periphery of breast

Potential for wide spread use	Fair	Very good	Very good	Very good	Very good	Very good	Very good	Very good	Limited	limited	fair

Clinical outcome data	11 years case studies	5 years case studies	None	Limited	Limited	None	4.5 years case studies	8 years case studies	Limited	4 years Case studies	4 years RCT

Main drawback	High expertise required and QA	Stringent QA is required Cavity, shape and size	Cavity shape and size	Cavity shape and size	Treatment planning complex	Treatment planning complex	Setup and breathing errors	High skin Dose	Expensive and 2^nd ^neutrons	Pathology not available	Pathology not available

### Patient Selection

Patient selection is critical to the successful application of APBI[136]. In a recent review, Polgar et al. [137] argued that the relatively poorer results of early APBI studies, with high local recurrence rates exceeding 1% per year could be attributed to inadequate patient selection criteria and/or suboptimal treatment technique and lack of appropriate QA procedures. Similarly in a recent study by Chen et al. [76], 70 patients were treated with MammoSite at the median follow up of 26.1 months, four local failures were observed of which two did not meet the ABS and ASBS selection criteria. These failures highlight the need to better define the subset of patients for whom APBI is most appropriate. Various societies have now published recommendations of patient selection criteria for APBI. These include, the American Society of Breast surgeons (ASBS), the American Brachytherapy Society (ABS), American Society for Radiation Oncology (ASTRO) and European Society for therapeutic Radiology and Oncology (ESTRO) [48,137,138]. The recent GEC-ESTRO recommendations ([137] have stratified the patients into three groups: low risk, intermediate and high risk (contraindication for APBI); similarly, ASTRO [138] has stratified them into suitable, cautionary and unsuitable. The low risk (suitable) group describes patients where APBI outside of a clinical trial would be considered acceptable (see Table 6); these criteria are stricter than those recommended by the ASBS or ABS. However, less restrictive criteria could be applied to patients who enrolled in a clinical trial. Generally young patients (< 50 years) and those who may harbor disease a significant distance from the edge of the excision cavity or potentially have multi-centric disease should not be treated with APBI off protocol. It also worth noting that these recommendations were determined from a systematic review of the APBI literature. The groupings were based primarily on an analysis of the characteristics of patients most frequently included in trials of APBI and not on data that identified subsets of patients with higher rates of ipsilateral breast tumor recurrence (IBTR) when treated with APBI. Recent analysis using ASBS registry trial [139,140] and using data from using of University of Wisconsin [141] show that the ASTRO consensus groupings may not be optimal in identifying patients for APBI.

**Table 6:** ASTRO and GEC-ESTRO suitable patient recommendation selections for APBI outside of clinical trials

	**Suitable group by ASTRO **[138]	**Low Risk group by GEC-ESTRO **[137]
**Factors**	**Criterion**	**Criterion**

Age	> 60 y	> 50

BRCA 1, 2 Mutation	Not present	na

Tumor Size	< 2 cm	< 3 cm

T stage	T1	T1-2

Margins	Negative by at least 2 mm	Negative by at least 2 mm

Grade	any	any

LVSI	Not allowed	Not allowed

ER status	positive	any

Multicentricity	unicentric	unicentric

Multifocality	Unifocal with total size of < 2 cm	unifocal

Histology	IDC, mucinous, tubular and colloid	IDC, mucinous, medullary, colloid

DCIS	Not allowed	Not allowed

EIC	Not allowed	Not allowed

Associated LCIS	Allowed	Allowed

Nodal status	pN0 (by SN Bx or ALND	pN0 (by SLNB or ALND)

Neoadjuvant Therapy	Not allowed	Not allowed

APBI = accelerated partial breast irradiation, IDC = invasive ductal carcinoma, ILC = invasive lobular carcinoma, LCIS = lobular carcinoma in situ; DCIS = ductal carcinoma in situ; EIC = extensive intraductal component; LVI = lympho-vascular invasion; ER = estrogen receptor; SLNB = sentinel lymph node biopsy; ALND = axillary lymph node dissection

### Fractionation Scheme

One concern regarding APBI is the proliferation of approaches; this inherently makes it difficult to elucidate the generic effect of APBI from the specific effect of a particular technique. As described within this review, many dosing schemes have been used; the different fractionation schemes and different BED make it possible for a failure to occur due to inappropriate dosing rather than the fact that only a partial region of the breast had been irradiated using APBI. Taking the 3D-CRT approach as an example, (see Table 3) many dosing schemes have been reported; for example, in the ELIOT studies, three different dose levels were used: 20 Gy (seven patients), 22 Gy (20 patients), and 24 Gy (20 patients) [132]. Further, the use of soft x-rays as in Intrabeam and Xoft approaches introduce another concept of relative biological effectiveness; thereby introducing another variable when trying to determine the effectiveness of the dosing.

### Target Definition

The basic tenet of radiation therapy is the delivery of a tumorcidal dose to the clinical target volume. In terms of applying APBI, there are questions of the appropriate target volume; is 1 cm or 2 cm enough margin for the irradiation of residual tumor? Depending on the particular technique, the delineation of this target can be problematic. It has been well documented that inappropriate target delineation will result in under dosing of the tumor or irradiating excessive volumes of normal tissues and organ at risk [142]. For non-brachytherapy techniques, substantial differences in delineation of the lumpectomy cavity have been observed, even by dedicated breast radiation oncologists [98]. The definition of the CTV is influenced by clinical features in the breast such as dense breast parenchyma, benign calcifications, low seroma clarity score, small volume and proximity to the pectoralis muscles [143]. To facilitate the contouring, surgically placed clips after lumpectomy have demonstrated strong radiographic surrogates of the lumpectomy cavity [99,144]. Also, written guidelines for contouring CTV have been shown to significantly reduce the inter-observer variability and minimize the volumes for radiation [145].

### Clinical Trial Evidence

As seen in tables 1, 2, 3, 4, the longest follow-up for APBI is with multi-catheter interstitial brachytherapy (MIB). Vicini et al. [146] have shown local recurrence rates of 3.6% at 10 years. More recently, Polgar et al. [58] reported a 12 year prospective study using MIB; four (8.9%) ipsilateral breast tumor recurrences were observed, for a 5-, 10- and 12 year actuarial rate of 4.4%, 9.3% and 9.3% respectively. The other techniques have shorter follow-up, with no local recurrence rates at 5 years follow up for MammoSite brachytherapy (MSB) [73], no reported recurrences in 10 to 28 months with single institution studies of 3D-CRT and 1.3% at 19 months in a single-institution study of intra-operative radiation therapy (IORT) [147].

It is now accepted that critical evaluation of clinical studies is appropriately done in terms of evidence based medicine. There are a few methodologies for reviewing the quality of the evidence including SORT (strength of recommendation taxonomy)[148], Grade (grades of recommendation, assessment, development and evaluation)[149] and CEBM (center for evidence based medicine). This critical evaluation is usually done under the umbrella of a systematic review and meta-analyses. The present review was not designed as a systematic review, but as a detailed analysis focussed towards providing the details and nuances of the different techniques. Nonetheless, an evaluation of a particular technique will not be complete without some assessment of its clinical validity. In terms of the SORT approach of clinical evidence, RCT provides level 1 clinical evidence of efficacy and validity. There are currently four reported APBI RCT [63,123,150,151]. However, not all of these studies met the SORT recommendation of quality, quantity and consistency. The YBCG (Yorkshire Breast Cancer Group)[151] and Christie Hospital[150] trials lack consistency in terms of patient selection and appropriate target definition, So, these two trials only provides level 3 evidence of efficacy. The Hungary trial [63] lacks the sample size to detect a difference. Hence, the only RCT that provides level 1 evidence is the recently published TARGIT study[123]. Case control studies provide level 3 evidence of efficacy and validity. Hence, for the other APBI techniques (MIB, Mammosite, 3DCT, and electron IORT) there is currently only level 3 evidence of efficacy.

Hence clinical community awaits the results of the other ongoing trials for more data on the long-term effectiveness of these techniques. Seven current phase III randomized clinical trials are currently evaluating the clinical efficacy of these APBI techniques (see Table 7); these studies include the National Surgical Adjuvant Breast and Bowel Project (NSABP) B- 39/Radiation therapy oncology group (RTOG) 0413 trial, RAPID(randomized trial of accelerated partial breast irradiation)/Ontario clinical oncology group, GEC-ESTRO, IMPORT-LOW (intensity modulated and partial organ radiotherapy) trial in the UK, electron intra-operative therapy (ELIOT) trial and targeted intra-operative radiotherapy (TARGIT) [52].. These trials have been examined in great details recently by Mannino and Yarnold [27] identifying the differences between them. These trials differ in patient selection criteria, radiotherapy technique used in the experimental (APBI) arm, radiation dose and fractionation scheme [27,152]. The sample size also varies with the different trials. If one was to assume an annual recurrence rate of 1% or less and a randomization ratio of 1:1, one will need about 810 patients per arm, assuming no attrition in three years (80% power and 95% confidence). All the trials met this requirement apart from the ELIOT trial. A larger sample size as required in the NSABP trial increases the power to detect smaller differences. In addition, a larger sample size makes it possible to study subgroups of patients with statistical power to detect a difference. If these trials accrue to target, almost 16000 women will be followed, hence providing level I evidence for or against the application of APBI in women with early stage breast cancer.

**Table 7:** Prospective randomized phase II clinical APBI adapted from Offersen et al. [52] and Lehman and Hickey [152] WBI = whole breast irradiation, RAPID = randomized trial of accelerated partial breast irradiation

Trial	Trial Design	N	Inclusion	Control Arm	APBI technique (Experimental Arm)	Status
TARGIT [123]	Equivalence	2232	≥ 45 years T1 small T2, N0, 1, Ductal	WBI as per institutional guidelines	IORT, Low energy X-rays 50 KV, 20 Gy/1 fraction	Started March 2000, completed enrollment march 2010

ELIOT [115]	Equivalence	824	≥ 48 years invasive carcinoma T ≤ 2.5 cm, pN0, Quadrantectomy	WBI, 50 Gy/25 fractions + optional 10 Gy Boost	IORT 21 Gy/1 fraction, electrons up to 9 MeV	Started in Dec 2000

GEC-ESTRO	Non-inferiority, non-irrelevant, 3% difference	1170	≥ 40 years stages O-II ductal/lobular carcinoma T ≤ 3 cm, pNO-pNmi, margin ≥ 2 mm	WBI 50- 50.4 Gy/25-28 fractions + optional 10 Gy boost	MIB, 32 Gy/8 fractions HDR, 30.3 Gy/7 fractions HDR, 50 Gy PDR	Started 2004

NSABP/RTOG 0413	Equivalence	4300	≥ 18 years stage 0, I, II (T < 3 cm) DCIS or invasive adenocarcinoma, ≤ 3 nodes positive, Margin negative	WBI 50-50.4 Gy/25-28 fractions, optional 10- 16 Gy boost	MIB Mammosite 34 Gy/10 fractions (5-10 days) 3D EBCRT 38.5 Gy/10 fractions (5-10 days)	Started in 2005 (accrual now closed to low risk patients)

RAPID	Equivalence	2128	≥ 40 years DCIS or invasive carcinoma T< 3 cm, margin negative, node negative, not BRCA 1/BRCA 2	WBI 42.5 Gy/16 fractions/22 days (small breast) 50 Gy/25 fractions/35 days (large breast plus optional boost 10 Gy/4-5 fractions	3D CRT 38.5 Gy/10 fractions (5-8 days) Minimum daily fraction separation 6 -8 hours	Started in January 2006

IMPORT-LOW	Non-inferiority	1935	≥ 50 years invasive adenocarcinoma (not lobular) T ≤ 3 cm, margin ≥ 2 mm, node negative	WBI 40 Gy/15 fractions/21 days	EBRT (IMRT) Arm 1 40 Gy/15 fractions to primary tumour region + 36 Gy/15 fractions to low risk region Arm 2 40 Gy/15 fractions to primary tumour region	Started in 2006

IRMA	Non-inferiority	n/a	≥ 49 years pT1-2 (< 3 cm) invasive carcinoma pN0- N1 Margins ≥ 2 mm	WBI 45 Gy/18 fractions, or 50 Gy/25 fractions, or 50,4 Gy/28 fractions	3D CRT 38.5 Gy total in 10 fractions (3.85 Gy per fraction), twice a day with an interval of at least 6 hours	Started in 2007

### APBI in Asia

Breast Conservation Therapy (BCT) in the Asia region has not observed the level of interest and growth observed in the western countries. In Hong Kong, the limited usage of BCT has been associated with limited number of radiation therapy facilities [154]. However, because of the increasing local experience in the administration of BCT, increasing numbers of young patients in the population and increasing efforts to promote breast cancer awareness in recent years, the use of BCT is steadily increasing [154]. For example, in Western Australia the proportion of women under going initial BCT doubled from 33% in 1982-1985 to 72% in 1998-2000 [155]. One will further expect that APBI to increase the use of BCT in the management of early breast cancer. However, there is another issue in the application of APBI to the Asian population which is breast size. Asian women generally have smaller breast compare to European. Some of the APBI techniques might be challenging to apply to this patient group. In Japan for example, excision involving 2 cm free margin from the tumor is most commonly performed. In many cases mammary gland tissue does not remain on the dermal or pectoralis muscle sides of the tumor. The target of irradiation is only the lateral stump [23]. Hence APBI techniques like the Mammosite will not be very applicable in the Asian population, because of potential excessive radiation dose to the skin. Maybe more conformal techniques like the SAVI or Clearpath might be appropriate. However, treatment results have not yet been published.

3D-CRT APBI also has similar limitations in the Asia region. When irradiation is performed in the supine position, flat extension of the breast reduces the distance between the target of the irradiation and the skin, leading to excessive exposure of the skin. However, using the 4field technique of 3D-CRT, Kosata et al. [156] demonstrated that in Japanese women, patients with a laterally located small tumor can be candidates for APBI, although patients with medially located tumor cannot. They also noted that a new beam arrangement using a combination of photons and electrons (a three-field technique that consisted of opposed, conformal tangential photons and enface electrons) recently proposed by Massachusetts General Hospital [101] may be more suited to Japanese women than that of the NSABP B-39/RTOG 0413 protocol [156].

IORT is also been explored as a way to provide APBI to the Japanese population. A phase I study designed using a scheme of dose-escalation from 19, 20, and 21 Gy at 90% isodose has been reported by Sawaki et al.[157]. The IORT treatment was well tolerated in Japanese women, with a prescription dose of 21 Gy was recommended.

### Dose Coverage

The coverage of the target varies depending on the technique. There are limited studies evaluating multiple techniques [158]. Weed and colleagues compared 3D-CRT, mammosite and interstitial brachytherapy; they found that at the coverage at 90% of the prescribed dose, no difference was observed between 3D-CRT and MammoSite (which were both better than interstitial)[158]. 3D-CRT resulted in better coverage of the PTV compared with MammoSite or interstitial brachytherapy techniques. Better PTV coverage with 3D-CRT came at the cost of a higher integral dose to the remaining normal breast. Dosimetrically, the best partial breast irradiation technique appears to depend on the clinical situation.

### Quality of Life (QOL)

In addition to local control, improved survival and better cosmesis, quality of life is also an important variable in evaluating treatment technique for breast cancer patients; with limited studies evaluating QOL aspects of breast cancer treatment. In a study by Wadasadawala et al [159], comparing APBI and WBI they found that the scores for social functioning and financial difficulties showed a trend towards a better outcome in the APBI group (p = 0.025 and p = 0.019 respectively). However, body image was significantly better in the APBI group as compared with the WBRT group (p = 0.005). Reports evaluating QOL for the different APBI have not been reported to date; although patients undergoing Mammosite have been reported to be very satisfied with their outcome [160].

### Cost Effectiveness

In the age of rapidly increasing health care costs, evaluation of techniques has to include cost effectiveness. Cost comparisons have been reported by Suh et al. [161,162] and Sher et al. [163] modeled treatment planning and delivery for different WBI fractionation schemes, Mammosite, MIB, APBI - 3DCRT and APBI-IMRT. They found that the least expensive partial breast-based radiation therapy approaches were the external beam techniques (APBI-3D-CRT and APBI-IMRT); any reduced cost to patients for the HDR brachytherapy-based APBI regimens were overshadowed by substantial increases in cost to payers, resulting in higher total societal costs. The cost of HDR treatment delivery was primarily responsible for the increased direct medical cost. APBI approaches in general were favored over whole-breast techniques when only considering costs to patients. However, If one were to pursue a partial-breast radiation therapy regimen to minimize patient costs, it would be more advantageous from a societal perspective to pursue external beam-based approaches such as APBI-3D-CRT or APBI-IMRT in lieu of the brachytherapy-based regimens [162]. Similarly, Sher et al. [163] reported that APBI-3DCRT was the most cost-effective strategy for postmenopausal women with early-stage breast cancer. Unless the quality of life after MSB proves to be superior, it is unlikely to be cost-effective [163].

### Further Research

As eluded in this review, there are still a few unanswered questions including optimal technique, patient selection and target volume definition.

#### 1 Optimal Technique

As reviewed herein, there are quite a variety of techniques available for APBI, but with insufficient clinical and dosimetric data to determine the optimal technique. It is worth noting that none of the current RCT will address this issue since a direct comparison of the technique is not part of any of the current trials. So research is required to determine (a) what is the optimal technique? (b) what technique is best for which patient?. Breast size and location of the lumpectomy cavity might dictate which technique to use. For example, small breasted patients might be best suited for IORT, while larger breasted are best served by balloon based brachytherapy techniques such as the MammoSite.

#### 2 Patient Selection

There is yet to be a consensus in terms of which patients characteristics are suitable for APBI. Different societies have come up with varying patient selection criteria. Current data analysis shows that these recommendations might not be optimal. Therefore there is a need for a definitive clinical and pathological criteria for APBI patient selection.

#### 3. Target Volume Definition

As reviewed herein, the volume of breast tissue irradiated varies with the technique used. Empirical and pathological studies are required to determine the level and degree of spread of micro-calcification. This will give a definitive guidance on how much tissue needs to be irradiated.

#### 4 Optimal Dose and Fractionation Scheme

There is growing evidence that the linear quadratic model (LQM) may not be appropriate for modeling high dose per treatment[164,165]. It has been suggested that LQM consistently overestimates cell killing at high single doses because it predicts a survival curve that continuously bends downward, whereas the experimental data are consistent with a constant slope (D0) at high doses. Furthermore, high-dose radiotherapy is achieving higher local control than could be explained by our current knowledge of radiation killing of cancer cells in a tumor. Proper radiobiological modeling is required to determine the optimal dose for APBI and fractionation scheme for the different techniques. The impact of the radiation energy used in determining the dosing also has to be investigated. For example, the dose for low energy x-rays will be different from the dose needed for high energy x-rays.

#### 5. Imaging and Pathology

The role of imaging in the management of most diseases is unquestionable. This is also true for breast cancer management. The success of APBI depends highly on the ability to identify patients at low risk of multi-centric disease. Hence, the appropriate imaging technique has to be determined. For example, the value of adding a preoperative breast MRI to conventional mammography remains controversial[166]. Hence, an imaging technique is required to increase the specificity and sensitivity of multi-centric disease diagnosis.

## Conclusions

The interest in APBI is evident from the proliferation of approaches and devices. However, studies are required, not only to evaluate the efficacy of APBI, but also to assess the safety and toxicity of the various techniques and dosing schedules. Furthermore, it is hoped that more research will be carried out to determine the strengths and weaknesses of the different techniques; thereby creating a consensus and identifying where each technique may be best applied. Whole Breast Irradiation (WBI) as part of Breast Conservation Therapy has well established results in terms of disease control, good cosmesis, and low toxicity. The acceptance of APBI as a standard of care therefore rides on its ability to match or better WBI in terms of efficacy, quality of life outcomes, and cost-effectiveness.

## Competing interests

The authors declare that they have no competing interests.

## Authors' contributions

CFN, MWS, CML: conception and design. CFN drafted the manuscript, MWS and CML critiqued the manuscript. CFN, MWS and CML read and approved the final manuscript.
